# Assessment of Iron Deficiency Anemia in Children Aged 6–24 Months at the Follow-Up Clinic of Abia State Children's Specialist Hospital, Umuahia, Nigeria: A Cross-Sectional Study

**DOI:** 10.7759/cureus.89971

**Published:** 2025-08-13

**Authors:** Chimaobi Ezekiel Ijioma, Izuchukwu E Okeji, Chiemela Obiocha Onubogu, Oladoyin Ogunbayo Jolaoye, Ngozi Uloma Enwereji, Christopher C Okafor, Olalekan E Aminu-Ayinde, Ugonna Emmanuella Ojumonu, Ihechiluru Lazarus Nwokeafor, Uzoma Ndukwe, Chinenye Adanna Ngwogu, Kelechi Kenneth Ngwogu, Nelson Obioma Uzor, Peace D Akhimienmhona, Prosper Chisom Okebugwu, Chukwudi Jeffrey Ekweozor

**Affiliations:** 1 Pediatrics, Abia State Children's Specialist Hospital, Umuahia, NGA; 2 Pediatrics, University of Arkansas for Medical Sciences, Little Rock, USA; 3 General Medicine, North Cumbria Integrated Care, NHS Foundation Trust, Cumbria, GBR; 4 Internal Medicine, Abia State University Teaching Hospital, Aba, NGA; 5 Internal Medicine-Pediatrics, University of Illinois College of Medicine at Peoria/OSF Saint Francis Medical Center, Peoria, USA; 6 Medicine, Ebonyi State University, Abakaliki, NGA; 7 General Medicine, Comfort Clinic, Ibafo, NGA; 8 Internal Medicine, Federal Medical Centre, Umuahia, NGA; 9 Internal Medicine, Nigerian Christian Hospital, Onicha Ngwa, NGA; 10 Neurology, University Hospitals Coventry and Warwickshire, Coventry, GBR; 11 Community Medicine, Abia State University Teaching Hospital, Aba, NGA; 12 Pediatrics, University Hospital, Southampton, GBR; 13 Internal Medicine, V. N. Karazin Kharkiv National University, Kharkiv, UKR; 14 Family Medicine, Hennepin Healthcare, Minneapolis, USA; 15 Internal Medicine, University College Hospital, Ibadan, NGA

**Keywords:** caregiver awareness, children under two, dietary diversity, hemoglobin, iron deficiency anemia, maternal education

## Abstract

Background

Iron deficiency anemia (IDA) is a major public health concern among children under two years of age, particularly in developing countries such as Nigeria. During this critical period of growth and brain development, undiagnosed and untreated IDA can lead to long-term cognitive, behavioral, and physical impairments. Timely identification and intervention are therefore essential to improving health outcomes and reducing the burden of childhood anemia.

Aim

The aim of the study was to assess the prevalence, severity, and associated factors of IDA among children aged 6-24 months attending the follow-up clinic at Abia State Children's Specialist Hospital, Umuahia, Nigeria.

Methods

This hospital-based cross-sectional study involved 215 systematically selected children aged 6-24 months. Data were collected using structured interviewer-administered questionnaires covering sociodemographics, feeding practices, medical history, caregiver knowledge, and healthcare-seeking behavior. Anthropometric measurements followed WHO guidelines. Hemoglobin concentration, serum ferritin, and C-reactive protein (CRP) levels were measured. IDA was defined as hemoglobin <11.0 g/dL and serum ferritin <12 µg/L, or <30 µg/L with elevated CRP. Data were analyzed using IBM SPSS Statistics for Windows, version 20 (IBM Corp., Armonk, NY), employing chi-square tests, bivariate analysis, and multivariable logistic regression. Statistical significance was set at p<0.05.

Results

The prevalence of IDA was 13.6%, while the overall anemia prevalence was 37.7%. Among anemic children, 19.1% had mild, 14.0% had moderate, and 4.7% had severe anemia. Microcytic hypochromic anemia accounted for 59.3% of cases. Significant predictors of IDA included low dietary diversity (adjusted odds ratio (AOR)=2.42; 95% confidence interval (CI): 1.17-4.99; p=0.017), maternal education ≤ primary level (AOR=1.98; 95% CI: 1.03-3.79; p=0.041), and household income <₦30,000 (AOR=2.11; 95% CI: 1.09-4.10; p=0.027). Caregiver knowledge was poor in 28.8%, and only 36.7% reported prior use of iron supplements.

Conclusion

IDA remains a notable concern among children aged 6-24 months in Umuahia, driven by socioeconomic and nutritional factors. Strengthening caregiver education, promoting dietary diversity, and encouraging early screening are essential to reducing the burden of IDA in this vulnerable population.

## Introduction

Iron deficiency anemia (IDA) remains a major global public health concern, particularly affecting infants and young children [1]. According to the World Health Organization, approximately 27% of the global population is anemic, with iron deficiency accounting for nearly half of these cases. Moderate-to-severe IDA impacts about 8.8% of the population, including 15% of children aged 1-3 years [2]. In sub-Saharan Africa, up to 60% of anemic children are under five years old, with iron deficiency being the predominant cause [3].

In Nigeria, anemia affects an estimated 68% of children under five years of age, as reported in the 2018 Nigeria Demographic and Health Survey, a prevalence categorized as high [4]. While multiple factors contribute to anemia in this age group, such as malaria, helminth infections, and hemoglobinopathies, dietary iron deficiency remains the most common cause. Over 50% of anemia cases in young children are attributed to inadequate iron intake [5]. The age range of 6-24 months is especially vulnerable due to rapid growth demands and the depletion of neonatal iron stores, necessitating adequate dietary intake or supplementation during this period [5]. Iron deficiency during infancy, even in the absence of anemia, has been associated with long-term impairments in cognitive, motor, and behavioral development. Because breast milk contains limited iron, exclusively breastfed infants are particularly vulnerable after 4 to 6 months of age, underscoring the importance of timely supplementation [6,7].

Regional studies in Nigeria have reported varying prevalence rates and identified risk factors for IDA. For instance, Fajolu et al. [8] reported a 14.9% prevalence among children aged 6-24 months in Lagos, with low intake of animal protein and vegetables as key contributors. Similarly, Akodu et al. [2] found a 10.1% prevalence of IDA among preschool children and highlighted underweight and wasting as significant predictors.

National-level surveys have pointed to additional sociodemographic risk factors. Children aged 6-12 months are at particularly high risk of anemia, while maternal education and household income have been shown to be protective [9]. Malnutrition, which manifests as stunting, wasting, or underweight, also increases susceptibility to IDA, reinforcing the multifactorial nature of its determinants [4].

Despite the growing body of evidence, most studies have focused on urban centers such as Lagos or used broad national datasets. Hospital-based data from secondary cities such as Umuahia, Abia State, remain limited. Yet, pediatric referral centers in these regions not only provide care to a significant portion of the population but also offer valuable insights into the burden, severity, and drivers of IDA. Furthermore, understanding caregiver knowledge and health-seeking behavior is crucial, as these factors influence early recognition and intervention.

Against this backdrop, the present study aims to address this gap by assessing the prevalence and severity of IDA, identifying associated risk factors, and evaluating caregiver knowledge and health-seeking practices among children aged 6-24 months attending a follow-up clinic at Abia State Children's Specialist Hospital, Umuahia, Nigeria.

## Materials and methods

Study design

This hospital-based cross-sectional descriptive study was conducted to determine the prevalence, severity, and associated factors of IDA among children aged 6-24 months. The study also evaluated predictors of IDA and assessed caregiver awareness and health-seeking behavior.

Study area

The study was carried out at Abia State Children's Specialist Hospital, Umuahia, a pediatric referral facility offering outpatient, inpatient, immunization, nutritional counseling, and follow-up care services for children under five years of age. It serves both urban and rural communities in and around Umuahia, the capital of Abia State, Nigeria.

Study setting

The study was carried out at the outpatient clinic at Abia State Children's Specialist Hospital between November 2024 and May 2025.

Study participants and eligibility criteria

Participants were children aged 6-24 months attending the follow-up and general outpatient pediatric clinics, along with their caregivers at Abia State Children's Specialist Hospital, Umuahia. Caregivers provided written informed consent before participation.

Inclusion criteria

The study included children aged 6-24 months attending the hospital's follow-up or general outpatient clinic whose caregivers provided informed consent and who had no chronic disease or congenital anomaly.

Exclusion criteria

Children were excluded if they had diagnosed hematological disorders (e.g., sickle cell disease and thalassemia), a history of blood transfusion within the last three months, were preterm or of low birth weight (<2500 g), were receiving iron-fortified formula from birth, or if their caregivers refused participation.

Sample size determination

The sample size for this study was calculated using the Cochran formula for estimating proportions in a population, as outlined by Ijioma et al. [10]. The formula used was

$n = \frac{Z^2 (Pq)}{e^2}\text{}$

where n represented the minimum sample size, Z was the Z-score at a 95% confidence level (1.96), P was the known prevalence of IDA in children under two years in Nigeria, e was the margin of error tolerated at 5% (0.05), and q was equal to 1 - P.

A study conducted by Fajolu et al. [8] reports the prevalence of IDA in children under two years in Nigeria as 14.9% (P=0.149), making q equal to 0.851.

$n = \frac{1.96^2 (0.149 * 0.851)}{0.05^2}\text{}$

resulting in a minimum sample size of 194.84, which was rounded up to 195. To account for a potential non-response rate of 10%, the sample size was further adjusted to 215.

Sampling technique

Systematic random sampling was used. Based on daily clinic attendance at Abia State Children's Specialist Hospital, every second eligible child was selected after a random starting point determined each day using a balloting method. Sampling continued daily until the target of 215 participants was reached.

Data collection instrument

A structured questionnaire developed by the authors was used to collect data, and the questionnaire was developed from various studies (Appendix) [4, 11-24]. It was pre-tested on a sample of 25 patients from a different hospital to assess its validity and reliability. Cronbach's alpha was used to assess the internal consistency of each section of the questionnaire. The feeding practices and medical history had a Cronbach's alpha of 0.74, the knowledge of caregivers section had 0.78, and health-seeking behavior of caregivers section had 0.78, indicating acceptable to good reliability across the components. The questionnaire covered the sociodemographic details, the feeding practices and medical history, the knowledge, and health-seeking behavior of the caregivers.

Anthropometric measurements

Measurements followed WHO protocols. Weight was measured using SECA® digital infant scale, calibrated daily, to the nearest 0.1 kg. Length was measured with infantometer to the nearest 0.1 cm. Mid-upper arm circumference (MUAC) was measured with a non-stretchable MUAC tape. Anthropometric indices (weight-for-age, height-for-age, weight-for-height, and MUAC) were computed using WHO Anthro software version 3.2.2 (WHO, Geneva).

Laboratory investigations

During sample collection, 3 mL of venous blood was collected aseptically into ethylenediaminetetraacetic acid and serum-separating tubes. Hemoglobin (Hb) was measured using an automated hematology analyzer (Sysmex® XN-1000, Sysmex Corporation, Kobe, Japan) . Serum ferritin and C-reactive protein (CRP) were measured using commercial enzyme-linked immunosorbent assay kits (MyBioSource MBS564042 for ferritin and a Rat CRP kit for CRP), with all procedures performed according to the manufacturers’ protocols. For inflammation adjustment, ferritin thresholds were adjusted based on CRP (>5 mg/L). For morphology, peripheral blood films were prepared and stained with Leishman stain; anemia type was classified microscopically by trained lab personnel. To maintain quality control, all samples were processed within two hours of collection. Internal controls and duplicate samples were used to ensure assay reliability.

Definitions and classifications

Anemia was defined as Hb concentration less than 11.0 g/dL and further classified as mild (10.0-10.9 g/dL), moderate (7.0-9.9 g/dL), and severe (<7.0 g/dL) [2]. IDA was defined as Hb <11.0 g/dL plus ferritin <12 µg/L (or <30 µg/L if CRP >5 mg/L) [25].

Assessment of knowledge and health-seeking behavior

Knowledge was assessed using a 5-point Likert scale (1 = strongly disagree to 5 = strongly agree) [26] and categorized as poor (1.0-2.4), moderate (2.5-3.9), and good (4.0-5.0). For health-seeking behavior, questions addressed promptness in seeking care, use of iron supplements, and preference for healthcare facilities vs. home/traditional remedies.

Data analysis

Data were analyzed using IBM SPSS Statistics for Windows version 20 (IBM Corp., Armonk, NY). Descriptive statistics (frequencies, means, and standard deviations) were used to summarize the data. Chi-square test and t-tests were used for bivariate analysis. Multivariable logistic regression was conducted to identify independent predictors of IDA. p-value of <0.05 was considered statistically significant.

Ethical considerations

Ethical approval for the study was obtained from the Ethics Committee of Abia State Children's Specialist Hospital, Umuahia, Nigeria, with approval number ABSCSH/EC/24/079, dated October 19, 2024. Written informed consent was obtained from all caregivers before their enrollment in the study. Participant confidentiality was ensured by assigning identification codes in place of names, and the data were stored securely. Participation was entirely voluntary, and participants retained the right to withdraw from the study at any time without any consequences.

## Results

A total of 215 children aged 6 to 24 months participated in the study. From Table 1 below, 72 (33.5%) children were aged 6-12 months, 80 (37.2%) were aged 13-17 months, and 63 (29.3%) were aged 18-24 months. The sample was almost evenly distributed by sex, with 108 (50.2%) males and 107 (49.8%) females. Regarding maternal education, 15 (7.0%) mothers had no formal education, 42 (19.5%) had completed primary education, 92 (42.8%) had secondary education, and 66 (30.7%) had attained tertiary education. In terms of maternal occupation, 91 (42.3%) were traders, 52 (24.2%) were civil servants, 38 (17.7%) were unemployed, and 34 (15.8%) were artisans or engaged in other occupations. Monthly household income distribution showed that 74 (34.4%) families earned less than ₦30,000, 83 (38.6%) earned between ₦30,000 and ₦60,000, and 58 (27.0%) earned above ₦60,000.

**Table 1:** Sociodemographic characteristics of children and caregivers

Variable	Frequency (n=215)	Percentage (%)
Child’s Age (Months)
6–12	72	33.5
13–17	80	37.2
18–24	63	29.3
Child’s Sex
Male	108	50.2
Female	107	49.8
Mother’s Education
No formal education	15	7.0
Primary	42	19.5
Secondary	92	42.8
Tertiary	66	30.7
Mother’s Occupation
Unemployed	38	17.7
Trader	91	42.3
Civil servant	52	24.2
Artisan/other	34	15.8
Monthly Household Income
	74	34.4
₦30,000–₦60,000	83	38.6
>₦60,000	58	27.0

From Table 2 below, among the 215 children studied, 142 (66.0%) were exclusively breastfed for the first six months. Complementary feeding was initiated at six months in 154 (71.6%) participants, before six months in 38 (17.7%) and after six months in 23 (10.7%). Dietary diversity, assessed using a 24-hour recall period, showed that 121 (56.3%) had high dietary diversity (consuming six or more food groups), 50 (23.3%) had moderate diversity (4-5 food groups), and 44 (20.4%) had low dietary diversity (fewer than four food groups). Regarding animal protein intake, 88 (40.9%) consumed animal protein daily, 98 (45.6%) occasionally (1-3 times per week), and 29 (13.5%) rarely or never. In the month preceding the study, 93 (43.3%) children experienced an infection and 38 (17.7%) had been hospitalized in the past six months. Deworming within the previous three months was reported in 139 (64.7%) children.

**Table 2:** Feeding practices and medical history (n=215)

Variable	Frequency	Percentage (%)
Exclusive breastfeeding (6 months)	142	66.0
Initiation of Complementary Feeding
<6 months	38	17.7
At 6 months	154	71.6
>6 months	23	10.7
Dietary Diversity (24-Hour Recall Period)		
Low (<3 food groups)	44	20.4
Moderate (4-5 food groups)	50	23.3
High (≥6 food groups)	121	56.3
Frequency of Animal Protein Intake
Daily	88	40.9
Occasionally (1–3x/week)	98	45.6
Rarely/never	29	13.5
Medical History
Recent infections (last 1 month)	93	43.3
Hospitalization history (past 6 months)	38	17.7
Dewormed in past 3 months	139	64.7

From Table 3 below, the majority of respondents demonstrated good knowledge regarding IDA in children under two years. A large proportion agreed (n=95, 44.2%) or strongly agreed (n=82, 38.1%) that iron is essential for blood formation and oxygen transport. Similarly, most participants acknowledged that IDA can affect a child’s physical and mental development, with 97 (45.1%) agreeing and 80 (37.2%) strongly agreeing. On the importance of early diagnosis and treatment, 95 (44.2%) agreed and 85 (39.5%) strongly agreed that timely intervention helps prevent long-term complications. In addition, 92 (42.8%) agreed and 60 (27.9%) strongly agreed that iron-rich complementary foods should be introduced after six months of age. Knowledge about sources of iron was also high. A total of 95 (44.2%) agreed and 78 (36.3%) strongly agreed that iron is found in both animal and plant sources, while 95 (44.2%) agreed and 87 (40.5%) strongly agreed that including vitamin C-rich foods helps improve iron absorption. Similarly, 92 (42.8%) agreed and 87 (40.5%) strongly agreed that regular health check-ups help in the early detection of iron deficiency. Only 68 (31.6%) agreed and 40 (18.6%) strongly agreed that exclusive breastfeeding for six months may not provide enough iron beyond that age, while 65 (30.2%) agreed and 40 (18.6%) strongly agreed that cow’s milk should not be a major part of an infant’s diet before 12 months due to its low iron content. Regarding contributors to anemia, 90 (41.9%) agreed and 65 (30.2%) strongly agreed that malaria and worm infestations can contribute to IDA in children. Furthermore, 90 (41.9%) agreed and 63 (29.3%) strongly agreed that iron supplements can help prevent or treat IDA. Awareness of the role of certain dietary inhibitors was moderate; 85 (39.5%) agreed and 50 (23.3%) strongly agreed that excessive tea or pap may reduce iron absorption, while 45 (20.9%) remained neutral.

**Table 3:** Caregivers’ knowledge of iron deficiency anemia Key: 1 = strongly disagree (SD), 2 = disagree (D), 3 = neutral (N), 4 = agree (A), 5 = strongly agree (SA), n = frequency, % = percentage.

Knowledge statement	SD		D		N		A		SA	
	n	%	n	%	n	%	n	%	n	%
I am aware that iron deficiency anemia is a common condition among children under 2 years.	15	7.0	18	8.4	30	14.0	87	40.5	65	30.2
I know that iron is an essential nutrient for blood formation and oxygen transport in the body.	6	2.8	10	4.7	22	10.2	95	44.2	82	38.1
I am aware that a lack of iron in the diet can lead to anemia in young children.	8	3.7	12	5.6	25	11.6	95	44.2	75	34.9
I know that exclusive breastfeeding for 6 months may not provide enough iron for babies beyond that age.	25	11.6	32	14.9	50	23.3	68	31.6	40	18.6
I am aware that iron-rich complementary foods should be introduced after the age of 6 months.	10	4.7	15	7.0	38	17.7	92	42.8	60	27.9
I know that cow’s milk should not be a major part of a baby’s diet before 12 months due to low iron content.	30	14.0	35	16.3	45	20.9	65	30.2	40	18.6
I am aware of the signs and symptoms of iron deficiency anemia (e.g., paleness, fatigue, and poor appetite).	14	6.5	18	8.4	30	14.0	85	39.5	68	31.6
I know that iron deficiency anemia can affect a child’s physical and mental development.	6	2.8	10	4.7	22	10.2	97	45.1	80	37.2
I understand that early diagnosis and treatment of IDA are important to prevent long-term complications.	5	2.3	10	4.7	20	9.3	95	44.2	85	39.5
I know that iron supplements can be given to children to prevent or treat iron deficiency anemia.	12	5.6	20	9.3	30	14.0	90	41.9	63	29.3
I am aware that malaria and worm infestations can contribute to anemia in children.	10	4.7	18	8.4	32	14.9	90	41.9	65	30.2
I know that iron can be found in both animal (e.g., liver and beef) and plant (e.g., spinach and beans) sources.	7	3.3	10	4.7	25	11.6	95	44.2	78	36.3
I understand that excessive tea or pap may reduce iron absorption in the body.	15	7.0	20	9.3	45	20.9	85	39.5	50	23.3
I believe it is important to include vitamin C-rich foods to enhance iron absorption.	5	2.3	8	3.7	20	9.3	95	44.2	87	40.5
I know that regular health check-ups help in the early detection of iron deficiency in children.	6	2.8	10	4.7	20	9.3	92	42.8	87	40.5

As shown in Table 4, the assessment of the level of caregivers’ knowledge revealed that 62 (28.8%) demonstrated good knowledge (≥75%), 91 (42.3%) had moderate knowledge (50%-74%), and 62 (28.8%) exhibited poor knowledge (<50%). When children showed symptoms suggestive of anemia, 134 (62.3%) caregivers reported seeking hospital care, while 81 (37.7%) relied on traditional or home remedies. Regarding iron supplementation, only 79 (36.7%) caregivers reported having previously given iron supplements to their children, whereas 136 (63.3%) had not.

**Table 4:** Knowledge level and health-seeking behavior of caregivers (n=215)

Variable	Frequency	Percentage (%)
Knowledge Level
Good (≥75%)	62	28.8
Moderate (50%–74%)	91	42.3
Poor (<50%)	62	28.8
Action for Suspected Anemia Symptoms
Hospital visit	134	62.3
Traditional/home remedies	81	37.7
Iron Supplement Use
Yes	79	36.7
No	136	63.3

As shown in Table 5, the nutritional assessment revealed that 42 (19.5%) children were underweight based on weight-for-age z-scores (WAZ), while 173 (80.5%) had normal weight-for-age. Stunting, assessed by height-for-age z-scores (HAZ), was observed in 46 (21.4%), with 169 (78.6%) classified as normal. Based on weight-for-height z-scores (WHZ), 39 (18.1%) children were wasted, whereas 176 (81.9%) had normal WHZ values. MUAC measurements indicated that 41 (19.1%) children had MUAC values ≤12.5 cm, suggestive of malnutrition, compared to 174 (80.9%) with normal MUAC values (>13.5 cm).

**Table 5:** Nutritional status based on anthropometry (n=215) HAZ, height-for-age z-score; MUAC, mid-upper arm circumference; WAZ, weight-for-age z-score; WHZ, weight-for-height z-score

Indicator	Category	Frequency	Percentage (%)
Weight-for-age (WAZ)	Normal	173	80.5
	Underweight	42	19.5
Height-for-age (HAZ)	Normal	169	78.6
	Stunted	46	21.4
Weight-for-height (WHZ)	Normal	176	81.9
	Wasted	39	18.1
MUAC	>13.5 cm (normal)	174	80.9
	≤12.5 cm (malnutrition)	41	19.1

**Figure 1:**
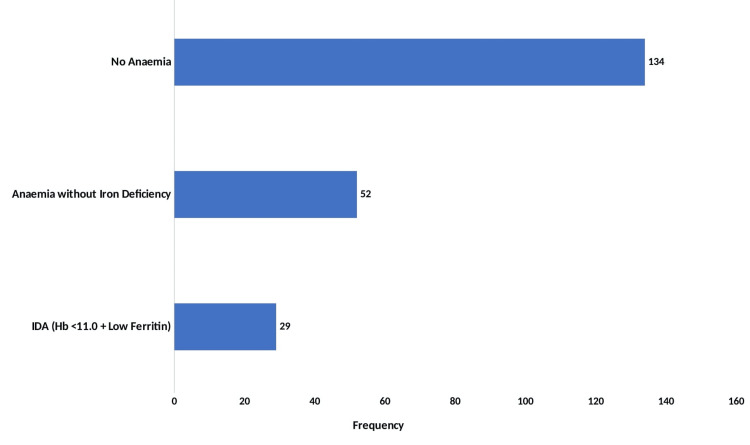
Prevalence of iron deficiency anemia Hb, hemoglobin; IDA, iron deficiency anemia

The findings presented in Figure 1 revealed that the overall prevalence of iron deficiency anemia was 29 (13.6%).

Of the 215 children assessed, as shown in Table 6, 134 (62.3%) were non-anemic, with Hb levels ≥11.0 g/dL. Mild anemia (Hb: 10.0-10.9 g/dL) was observed in 41 (19.1%), moderate anemia (Hb: 7.0-9.9 g/dL) in 30 (14.0%), and severe anemia (Hb: <7.0 g/dL) in 10 (4.7%) participants. Among children diagnosed with anemia, morphological classification revealed that 48 (59.3%) had microcytic hypochromic anemia, 24 (29.6%) had normocytic normochromic anemia, and 9 (11.1%) had macrocytic anemia.

**Table 6:** Severity and morphological classification of anemia (n=81 anemic children)

Variable	Frequency	Percentage (%)
Hemoglobin Status
Non-anemic (≥11.0 g/dL)	134	62.3
Mild anemia (10.0–10.9)	41	19.1
Moderate (7.0–9.9)	30	14.0
Severe (<7.0)	10	4.7
Type of Anemia (n)
Microcytic hypochromic	48	59.3
Normocytic normochromic	24	29.6
Macrocytic	9	11.1

Table 7 shows the relationship between key factors and the presence of IDA among children. Among children aged 6-12 months, 17.0% had IDA and 82.9% did not (p=0.021). Lack of exclusive breastfeeding was also associated with increased IDA prevalence (19.3% vs. 80.7%, p=0.012). Low dietary diversity was strongly linked to IDA, with 24.5% of children affected compared to 75.5% who did not (p=0.001). Additionally, children from households earning less than ₦30,000 had a higher rate of IDA (21.6%) than those from higher-income households (78.4%) (p=0.033). Maternal education at or below the primary level was similarly associated with increased IDA prevalence (22.7% vs. 77.3%, p=0.014).

**Table 7:** Bivariate analysis (sociodemographic associations with IDA) The chi-square (χ²) test was used to examine the association between categorical variables and IDA status. A p-value of <0.05 was considered statistically significant. IDA, iron deficiency anemia

Variable	IDA (%)	Non-IDA (%)	Test statistic (χ²)	p-value
Child age (6–11 months)	17.0	82.9	5.32	0.021
Exclusive breastfeeding (no)	19.3	80.7	6.35	0.012
Low dietary diversity	24.5	75.5	10.54	0.001
Household income	21.6	78.4	4.53	0.033
Maternal education ≤ primary	22.7	77.3	6.04	0.014

From Table 8 below, multivariable logistic regression identified several independent predictors of IDA among children aged 6-24 months (Table 7). Children with low dietary diversity had 2.42 times higher odds of IDA than those with adequate dietary diversity (adjusted odds ratio (AOR)=2.42; 95% confidence interval (CI): 1.17-4.99; p=0.017). Maternal education at or below the primary level was associated with nearly double the odds of IDA (AOR=1.98; 95% CI: 1.03-3.79; p=0.041). Household income below ₦30,000 also significantly increased the likelihood of IDA (AOR=2.11; 95% CI: 1.09-4.10; p=0.027), as did non-exclusive breastfeeding (AOR=1.88; 95% CI: 1.01-3.50; p=0.045). Although the younger age group (6-11 months) was associated with higher odds of IDA (AOR=1.71), this did not reach statistical significance (p=0.088).

**Table 8:** Multivariable logistic regression predictors of IDA Multivariable logistic regression was used to determine independent predictors of IDA. Wald chi-square (χ²) test statistic values are reported. A p-value of <0.05 was considered statistically significant. CI, confidence interval; IDA, iron deficiency anemia; OR, odds ratio

Predictor	Adjusted OR	95% CI	Test statistic (Wald χ²)	p-value
Low dietary diversity	2.42	1.17–4.99	5.66	0.017
Maternal education ≤ primary	1.98	1.03–3.79	4.17	0.041
Household income < ₦30,000	2.11	1.09–4.10	4.88	0.027
Non-exclusive breastfeeding	1.88	1.01–3.50	4.02	0.045
Child age 6–11 months	1.71	0.91–3.22	2.91	0.088

## Discussion

The results of our study provide critical insights into the assessment of IDA among children aged 6-24 months attending the follow-up clinic at Abia State Children's Specialist Hospital.

This study's sociodemographic profile revealed a fairly balanced distribution of children by age and sex, with the majority of participants aged between 6 and 17 months. The near-equal sex distribution (50.2% males and 49.8% females) mirrors findings from nationwide surveys indicating no significant sex bias in pediatric studies [11]. This suggests representativeness and reduces concerns about gender-related sampling bias. Maternal education levels showed that a significant proportion of mothers had secondary (42.8%) or tertiary education (30.7%), while 26.5% had primary education or no formal education. This is relatively higher than national averages reported by the Nigeria Demographic and Health Survey, which found that about 59% of women in similar age groups had only primary or no formal education [4]. Higher maternal education in this cohort could be attributed to the urban setting and access to healthcare services in Umuahia, which may influence health knowledge and behaviors positively [12]. Mother’s occupation data indicated a predominant involvement in trading (42.3%), with fewer mothers employed as civil servants (24.2%) or artisans (15.8%). Maternal occupation has been demonstrated to influence child nutrition and health outcomes through income stability and availability for childcare [13]. Regarding household income, over a third (34.4%) of families earned below ₦30,000 monthly, reflecting low socioeconomic status for a substantial portion of the sample. Low household income has consistently been linked to increased risk of IDA due to limited access to diverse and nutrient-rich foods [14].

In our study, 66% of children were exclusively breastfed for the first six months, which is significantly higher than the national average reported in the 2018 Nigeria Demographic and Health Survey, where exclusive breastfeeding among infants under six months was estimated at 29% [15]. This figure also surpasses the 16.4% reported in a study by Agho et al., suggesting that hospital-based populations may benefit from increased access to health education and follow-up support [16].

Regarding complementary feeding, 71.6% of caregivers initiated at six months, with 17.7% earlier and 10.7% later. This contrasts with the patterns described by Olatona et al., who reported 47.9% timely initiation and 16.0% dietary diversity in a Lagos cohort [17]. Dietary diversity over a 24-hour recall period showed that more than half (56.3%) of the children had high dietary diversity (≥6 food groups), which is a protective factor against IDA. Still, 20.4% of children had low dietary diversity (<3 food groups), placing them at higher nutritional risk. The role of dietary diversity in preventing micronutrient deficiencies, including IDA, has been widely documented. A study by Torlesse et al. in Indonesia showed that dietary diversity was strongly associated with Hb concentration and anemia prevalence in young children [18].

The frequency of animal protein intake showed that only 40.9% of the children consumed it daily. Although this is slightly above the 38%-42% regional estimates from studies across sub-Saharan Africa, it remains suboptimal [19]. Animal-source foods are key to improving iron intake and overall micronutrient adequacy in young children, and their low consumption has been consistently linked to increased risk of anemia and stunting [20]. Infectious morbidity was also common in this population, with 43.3% of children experiencing at least one recent infection in the past month and 17.7% having a history of hospitalization within the previous six months. Frequent infections can compromise iron absorption and dietary intake, further increasing the risk for IDA [21]. Encouragingly, 64.7% of the children had received deworming treatment in the past three months, a figure that is higher than the national average and likely reflects effective outreach through local health programs [4]. Our findings demonstrate that while infant feeding practices and health-seeking behaviors in this population show some strengths, especially in exclusive breastfeeding and deworming coverage, notable gaps persist in dietary quality and protein intake. These findings support previous evidence showing that maternal education, income, and healthcare access remain strong determinants of child nutrition outcomes [17,22].

This study assessed the knowledge of caregivers on IDA. Knowledge of the prevalence and consequences of IDA was encouraging. A total of 70.7% of respondents were aware that IDA is a common condition among children under two years. An even higher proportion, 82.3%, recognized that iron is essential for blood formation and oxygen transport, and 79.1% knew that insufficient dietary iron can lead to anemia in young children. Furthermore, 82.3% acknowledged that IDA can negatively impact a child’s physical and mental development, and 83.7% understood the importance of early diagnosis and treatment to prevent long-term complications. Respondents also demonstrated strong awareness of dietary contributors to iron status. A high proportion, 80.5%, correctly identified both animal and plant-based sources of iron. In addition, 84.7% believed in the importance of including vitamin C-rich foods to enhance iron absorption. On the role of healthcare in anemia prevention, 83.3% agreed that regular health check-ups are important for early detection. However, notable gaps were observed in knowledge about infant feeding practices and iron absorption inhibitors. Only 50.2% knew that exclusive breastfeeding may not provide enough iron beyond six months, and just 48.8% were aware that cow’s milk should not be a major part of a baby’s diet before 12 months. Knowledge of the need to introduce iron-rich complementary foods after six months was better, though still suboptimal at 70.7%. Understanding of factors that hinder iron absorption was moderate, with 62.8% acknowledging that excessive consumption of tea or pap can reduce iron absorption. Awareness of the signs and symptoms of IDA, such as paleness, fatigue, and poor appetite, was also moderate at 71.1%. Lastly, 72.1% of respondents correctly identified malaria and worm infestations as contributors to IDA, an important consideration in regions where such infections are endemic. Furthermore, 71.2% recognized that iron supplements can be used for the prevention and treatment of anemia in children.

In this study, the level of caregiver knowledge regarding IDA was suboptimal. Only 28.8% of caregivers demonstrated good knowledge, while 42.3% had moderate and 28.8% had poor knowledge scores. These results are consistent with findings from Onah et al., who reported similarly low levels of awareness on nutritional deficiencies among mothers in southeastern Nigeria, attributing it to limited formal education and low exposure to public health campaigns [23]. Limited knowledge has important implications for early detection and management of IDA. As shown in our findings, only 62.3% of caregivers reported taking their child to the hospital when they noticed symptoms suggestive of anemia, while a substantial 37.7% relied on traditional or home remedies. This pattern aligns with studies by Adegboyega et al., who highlighted that traditional or home remedies preferences often drive caregivers toward non-medical treatment options, delaying effective care [24].

Iron supplementation was reported by just 36.7% of respondents, indicating a gap in preventive practices. This underuse may reflect a lack of knowledge, limited access to supplementation programs, or poor adherence among caregivers. The reliance on hospital care by 62.3% is encouraging and slightly higher than national averages, suggesting improved health-seeking behavior in a hospital-based cohort [4]. However, the high proportion of caregivers relying on home remedies underscores the need for targeted community education.

The anthropometric analysis in our study population revealed that 19.5% of children were underweight based on WAZ, while 21.4% were stunted (HAZ) and 18.1% were wasted (WHZ). Additionally, 19.1% of children had MUAC <12.5 cm, consistent with moderate acute malnutrition. These findings are in line with national estimates from the 2018 Nigeria Demographic and Health Survey, which reported underweight prevalence of 22%, stunting at 37%, and wasting at 7% among under-five children [4]. However, the lower stunting rate (21.4%) observed in this study may reflect better access to healthcare and nutritional support among pediatric specialist hospital attendees.

The wasting rate of 18.1% in this study is notably higher than the national average. This may point to recent episodes of acute malnutrition or infection, particularly as 43.3% of the cohort reported recent illness. Wasting is often linked to inadequate dietary intake, recurrent infections, and poor feeding practices, which compromise short-term nutritional status [22].

The MUAC results aligned closely with WAZ and WHZ outcomes. About 19.1% of children had MUAC below 12.5 cm, classifying them as moderately acutely malnourished. MUAC is a practical field indicator for assessing acute malnutrition and is widely used in community and clinical settings for identifying at-risk children [27]. Compared with similar hospital-based studies in Nigeria, our findings are somewhat consistent. A study by Fajolu et al. in Lagos found a 20% prevalence of wasting and underweight among infants in urban clinics [8]. These comparisons suggest that while chronic malnutrition (stunting) may be lower in our sample, acute malnutrition remains a significant concern. Overall, these results highlight the dual burden of malnutrition, both chronic and acute, among children aged 6-24 months in Umuahia.

In our study, the prevalence of IDA (13.6%) among children aged 6 to 24 months is unprecedented in several pediatric settings in Nigeria. The investigation based in Lagos, for example, recorded a comparable percentage for IDA prevalence in the same age category (6-24 months) [8]. Yet, of a much higher nature are rates of IDA reported elsewhere, such as 16.1% for young children up to three years [28]. Globally, about 42% of anemia recorded in under-fives arise as a result of iron deficiency [5], thus signifying that the result found here corresponds with those reported locally, albeit a little below the country averages [4]. The composite prevalence of anemia (41.4%) equates to those of national rates. Child anemia has been reported to exist within the range of 46%-66% across sub-Saharan Africa, with Nigeria alone seeing child anemia rates of 68% within its under-five population [29]. Limiting the ages in our cohort from 6 months to 24 months covered the most critical risk periods of the younger children, especially the 6 to 12 months group, which supports earlier reports that anemia is highly prevalent in the younger infants [30].

The study revealed that 37.7% of children aged 6-24 months were anemic, with 19.1% having mild, 14.0% having moderate, and 4.7% having severe anemia, using WHO thresholds for Hb concentration [2]. This prevalence, while concerning, is lower than national estimates from the 2018 Nigeria Demographic and Health Survey, which reported an anemia rate of 68% among under-five children [4]. The lower prevalence in our study may be attributed to the hospital-based population, which typically has better access to medical care and nutritional advice than the general population. Similar rates were reported in a study by Fajolu et al. in Lagos, which found a 41.3% prevalence of anemia in children aged 6-24 months attending a tertiary hospital [8]. However, studies in rural areas, such as those by Uzochukwu et al., have reported significantly higher rates, indicating a clear urban-rural disparity in anemia burden and associated risk factors [31].

Morphologically, microcytic hypochromic anemia accounted for 59.3% of cases, confirming iron deficiency as the predominant cause in this population. This pattern is consistent with the well-established link between inadequate dietary iron intake and childhood anemia in low-income countries [21]. Normocytic normochromic anemia (29.6%) and macrocytic anemia (11.1%) were less common and may be associated with infections or deficiencies in folate or vitamin B12, respectively [32].

The bivariate analysis identified several sociodemographic and nutritional factors significantly associated with IDA in children aged 6-24 months. Children aged 6-11 months had a significantly higher likelihood of IDA than older children. This age group is particularly vulnerable due to rapid growth, depletion of prenatal iron stores, and the often-delayed introduction of iron-rich complementary foods. This trend mirrors findings from Pasricha et al., who reported that infants in this age range are at highest risk for IDA, especially in low-resource settings where dietary diversity is limited [32].

Non-exclusive breastfeeding was significantly associated with higher IDA prevalence. While exclusive breastfeeding is recommended for the first six months, breastmilk alone becomes insufficient to meet iron requirements after this period. Studies, such as by Chaparro and Dewey, support the need for timely and appropriate complementary feeding to prevent IDA in breastfed infants [33].

Children with low dietary diversity had significantly higher rates of IDA. This is consistent with findings from studies in Nigeria and other sub-Saharan African countries that link inadequate intake of animal proteins, fruits, and vegetables with iron deficiency [34]. Iron bioavailability from plant-based diets is low, and poor feeding practices exacerbate this risk.

Children from households earning less than ₦30,000 per month were more likely to have IDA. Socioeconomic disadvantage limits access to nutrient-rich foods and healthcare services. The 2018 Nigeria Demographic and Health Survey also identified low household wealth as a predictor of anemia among under-five children [4].

IDA was significantly more common among children whose mothers had only primary or no formal education. Low maternal education is a known determinant of poor infant feeding practices, delayed health-seeking behavior, and low utilization of preventive interventions. This association has been reported by Fajolu et al. in Nigeria [8]. These results highlight the multifactorial etiology of IDA, with nutrition, socioeconomic status, and caregiver education playing central roles.

The multivariable logistic regression analysis identified several significant independent predictors of IDA among children aged 6-24 months in this study. Children with low dietary diversity were over twice as likely to be iron deficient. This finding highlights the critical role of diet quality in preventing IDA and is consistent with studies conducted in South Asia, which showed strong associations between monotonous, plant-based diets, and anemia prevalence, especially in young children whose iron requirements are high due to rapid growth [21]. Although the global prevalence of dietary iron deficiency declined by 9.8% between 1990 and 2021, it remained substantial at 16.4%, an age-standardized rate of 16,434 per 100,000 population [35].

Maternal education was also significantly associated with child iron status. Children of mothers with no formal or only primary education had nearly double the odds of developing IDA. This underscores how caregiver knowledge, shaped by education level, influences feeding practices and healthcare decisions. Similarly, low household income (<₦30,000 per month) emerged as a strong predictor, suggesting that economic hardship limits access to nutrient-rich foods and preventive health services.

Non-exclusive breastfeeding was another significant factor. Children who were not exclusively breastfed for the first six months were more likely to be iron deficient. This supports WHO recommendations on exclusive breastfeeding, as breastmilk alone is adequate for iron needs in the first 4-6 months, but early or inappropriate complementary feeding may expose infants to iron-poor diets [36]. Although children aged 6-12 months had higher odds of IDA than older age groups, this association was not statistically significant. However, this trend is biologically plausible, as iron stores acquired in utero begin to deplete around six months, and many infants in this age group receive insufficient dietary iron [7].

Recommendations

Based on our findings of this study, several recommendations have been made to help reduce the burden of IDA among children in this vulnerable age group.

Strengthen Caregiver Education

Public health interventions should focus on improving caregiver knowledge of infant and young child feeding, the importance of exclusive breastfeeding, appropriate complementary feeding, and the early signs and consequences of anemia.

Promote Dietary Diversity

Nutrition programs should encourage the inclusion of a variety of locally available, iron-rich foods in the diets of infants and young children, especially from low-income households. Nutrition counseling should be integrated into routine child health visits.

Enhance Routine Screening and Supplementation

Health facilities should implement regular screening for anemia in children under two years, using Hb and iron status markers. Early identification should be followed by appropriate supplementation and management.

Improve Access to Affordable Nutrition

Social support programs should target economically disadvantaged households to improve access to iron-rich foods, either through subsidies or food fortification initiatives.

Sustain Deworming Campaigns

Given the role of helminths in contributing to iron loss, regular deworming of children should be maintained and strengthened, particularly in high-risk communities.

Conduct Longitudinal Studies

Further research using longitudinal designs is recommended to better understand causal relationships between feeding practices, socioeconomic status, and anemia progression, and to evaluate the long-term impact of interventions.

Limitation

Despite our efforts to provide a reliable examination of the study objectives, several limitations should be noted. As a cross-sectional study, the research offers only a snapshot in time, which restricts the ability to infer causal relationships between variables such as feeding practices, caregiver knowledge, socioeconomic factors, and the occurrence of IDA. Being hospital-based and conducted at a single pediatric referral facility also limits the generalizability of the findings to the broader population, especially children not attending health services. Data on feeding practices and health-seeking behavior were based on caregiver self-report, which may be influenced by recall bias or social desirability. While serum ferritin was used as a biomarker for iron status, it may be elevated in inflammatory states despite CRP adjustment, potentially affecting diagnostic accuracy. Furthermore, other contributors to anemia such as folate, vitamin B12 deficiency, or hereditary conditions were not assessed, which could lead to misclassification in some cases. In the future, increasing the sample size for the power of analysis will be helpful to have more generalizable results [37].

## Conclusions

This study shows the significant burden of IDA among children aged 6-24 months attending a pediatric referral hospital in Umuahia, Nigeria. Despite the majority of children receiving deworming treatment and demonstrating adequate dietary diversity, the prevalence of IDA remains concerning, suggesting that other underlying factors contribute to its persistence. The findings identify key predictors of IDA, including low maternal education, non-exclusive breastfeeding, poor household income, and inadequate dietary diversity in a subset of children. Additionally, caregiver knowledge about anemia and appropriate nutritional practices was often limited, pointing to a gap in health education. These results emphasize the need for comprehensive, community-based interventions focused on improving maternal health literacy, promoting exclusive breastfeeding, ensuring access to diverse and iron-rich foods, and strengthening early screening programs. Addressing these factors through coordinated public health strategies can play a pivotal role in reducing the incidence and long-term impacts of IDA among young children in similar settings.

## Appendices

**Table 9:** The questionnaire: assessment of iron deficiency anemia in children aged 6–24 months at the follow-up clinic of Abia State Children's Specialist Hospital, Umuahia, Nigeria: a cross-sectional study. Credits: Ijioma CE, Okeji IE, Onubogu CO, Jolaoye OO, Enwereji NU, Okafor CC, Aminu-Ayinde OE, Ojumonu UE, Nwokeafor IL, Ndukwe U, Ngwogu C, Ngwogu KK, Uzor NO, Akhimienmhona PD, Okebugwu PC, Ekweozor CJ.

Section	Question No.	Question	Response Options
Informed Consent	–	You are invited to participate in a research study titled Assessment of Iron Deficiency Anemia in Children Aged 6–24 Months at the Follow-Up Clinic of Abia State Children's Specialist Hospital, Umuahia, Nigeria. Participation is voluntary, responses are confidential, and you may withdraw at any time.	☐ I have read and understood the information and voluntarily agree to participate.
Sociodemographic Info	1	Age of the child (in months)	☐ 6–11 ☐ 12–17 ☐ 18–24
	2	Sex of the child	☐ Male ☐ Female
	3	Mother’s education level	☐ No formal education ☐ Primary ☐ Secondary ☐ Tertiary
	4	Mother’s occupation	☐ Unemployed ☐ Trader ☐ Civil Servant ☐ Artisan/Others: _______
	5	Monthly household income	☐ < ₦30,000 ☐ ₦30,000–₦60,000 ☐ > ₦60,000
Feeding & Medical History	6	Was child exclusively breastfed for first 6 months?	☐ Yes ☐ No
	7	When was complementary feeding introduced?	☐ Before 6 months ☐ At 6 months ☐ After 6 months
	8	Does child consume ≥4 food groups daily?	☐ Yes ☐ No
	9	Frequency of animal protein intake	☐ Daily ☐ Occasionally (1–3 times/week) ☐ Rarely/Never
	10	Infections in past 1 month (diarrhea, cough, fever)?	☐ Yes ☐ No
	11	Hospitalized in past 6 months?	☐ Yes ☐ No
	12	Dewormed in past 3 months?	☐ Yes ☐ No
IDA Knowledge	13	IDA is common among children under two years.	☐ 1 ☐ 2 ☐ 3 ☐ 4 ☐ 5
	14	Iron is essential for blood formation and oxygen transport.	☐ 1 ☐ 2 ☐ 3 ☐ 4 ☐ 5
	15	Lack of iron in the diet can cause anemia.	☐ 1 ☐ 2 ☐ 3 ☐ 4 ☐ 5
	16	Exclusive breastfeeding may not provide enough iron beyond 6 months.	☐ 1 ☐ 2 ☐ 3 ☐ 4 ☐ 5
	17	Iron-rich complementary foods should start after 6 months.	☐ 1 ☐ 2 ☐ 3 ☐ 4 ☐ 5
	18	Cow’s milk should be limited before 12 months due to low iron.	☐ 1 ☐ 2 ☐ 3 ☐ 4 ☐ 5
	19	I am aware of IDA symptoms (paleness, fatigue, poor appetite).	☐ 1 ☐ 2 ☐ 3 ☐ 4 ☐ 5
	20	IDA can affect a child’s physical and mental development.	☐ 1 ☐ 2 ☐ 3 ☐ 4 ☐ 5
	21	Early diagnosis and treatment of IDA are important.	☐ 1 ☐ 2 ☐ 3 ☐ 4 ☐ 5
	22	Iron supplements can prevent or treat IDA.	☐ 1 ☐ 2 ☐ 3 ☐ 4 ☐ 5
	23	Malaria and worm infestations can contribute to anemia.	☐ 1 ☐ 2 ☐ 3 ☐ 4 ☐ 5
	24	Iron is found in both animal and plant foods.	☐ 1 ☐ 2 ☐ 3 ☐ 4 ☐ 5
	25	Tea or pap may reduce iron absorption.	☐ 1 ☐ 2 ☐ 3 ☐ 4 ☐ 5
	26	Including vitamin C-rich foods helps improve iron absorption.	☐ 1 ☐ 2 ☐ 3 ☐ 4 ☐ 5
	27	Regular health checkups help detect IDA early.	☐ 1 ☐ 2 ☐ 3 ☐ 4 ☐ 5
Health-Seeking Behavior	28	Action if the child shows symptoms of anemia	☐ Take to hospital ☐ Use traditional/home remedies
	29	Do you give your child iron supplements?	☐ Yes ☐ No
Conclusion	–	Thank you for your time. For more information, speak with a healthcare provider.	—
